# Hip osteoarthritis signs and symptoms are associated with increased fall risk among community-dwelling older adults with chronic low back pain: a prospective study

**DOI:** 10.1186/s13075-021-02455-5

**Published:** 2021-03-03

**Authors:** Patrick J. Knox, Peter C. Coyle, Jenifer M. Pugliese, Ryan T. Pohlig, Jaclyn M. Sions, Gregory E. Hicks

**Affiliations:** 1 Department of Physical Therapy, University of Delaware, 540 S. College Ave, Newark, DE 19713 USA; 2 Department of Epidemiology, University of Delaware, Newark, DE USA; 3 Biostatistics Core, University of Delaware, Newark, DE USA

**Keywords:** Accidental falls, Aged, Low back pain, Osteoarthritis, Risk factors

## Abstract

**Background:**

Older adults with concurrent low back and hip pain are predisposed to reductions in physical performance and health-related quality of life. Yet no study to date has assessed whether or not coexisting hip impairments increase fall risk in older adults with chronic low back pain (CLBP). The objective of this study was to determine if hip osteoarthritis (OA) signs and symptoms per American College of Rheumatology (ACR) criteria are associated with fall risk over a 1-year span.

**Methods:**

Falls were prospectively monitored for 1 year via fall calendars. Age, sex, body mass index (BMI), anxiolytic use, balance confidence, LBP-related disability, and prior fall history were identified as covariates. Hip pain, pain with hip internal rotation (IR), hip IR range of motion (ROM) ≥ 15°, and morning stiffness lasting ≤ 60 min were evaluated at baseline and summed to represent hip OA impairment burden. A generalized linear model with a Poisson distribution and log link function assessed the association between ACR criteria and fall risk beyond established covariates. As a secondary analysis, binary logistic regression assessed ACR criteria and the odds of falling two or more times within a year.

**Results:**

Data from two-hundred and ten participants were analyzed. Hip OA signs and symptoms were present in 97.1% of the participants, and hip OA impairment burden was significantly greater (*p* < 0.050) in participants who fell ≥ 2 times compared to single and non-fallers. Higher hip OA impairment burden was associated with significantly increased fall risk (*p* = 0.001, risk ratio = 1.23, 95% CI 1.09–1.38) and odds of falling multiple times (*p* < 0.05, odds ratio = 1.41, 95% CI 1.01–1.95) after adjustment for covariates.

**Conclusions:**

Older adults with CLBP and concomitant hip impairments are an at-risk group for falling. Healthcare professionals should employ falls screening and preventive measures to avoid negative sequelae in this vulnerable population.

## Background

In the USA, low back pain (LBP) is the most frequently reported musculoskeletal complaint among older adults [1]. According to Medicare data from 1991 to 2002, there was a 131.7% increase in LBP patients and a 387.2% increase in LBP-related expenditures, suggesting that LBP is becoming more prevalent and costly among older adults [2]. Apparent increases in LBP prevalence among this age group are especially problematic because this clinical population is susceptible to steep functional decline [3, 4]. Consistent patient and public health burdens indicate that existing LBP management strategies are lacking; these concerns extend to chronic LBP (CLBP), which is understudied and poorly understood in older adults [5, 6]. Management of CLBP in older adults may be complicated by underlying influences from coexisting pain conditions [6, 7]; there is a need to explore how coexisting pain conditions, particularly hip impairments, may affect health outcomes in this clinical population.

Recent evidence suggests that coexisting hip impairments are more prevalent in and detrimental to older adults with CLBP. Hicks et al. [7] demonstrated that hip osteoarthritis (OA) signs and symptoms, as defined by American College of Rheumatology (ACR) criteria [8], are associated with poorer performance on physical function tests beyond the influence of CLBP alone; they are also associated with higher disability and reduced health-related quality of life in this patient population [9]. Rundell et al. [10] corroborate this association, as they report that older adults with coexisting hip OA and back pain had worse disability health-related quality of life than older adults with LBP alone. These findings are consistent with prior evidence on more distal health outcomes in older adults; hip OA has been established as a risk factor for falling and is associated with elevated annual fall prevalence compared to national estimates of falls in older adults [11, 12].

Falls are both costly and disabling for older adults [13], and individuals who fall two or more times within a year, or multiple fallers, are at greater risk of mobility decline, activities of daily living decline, hospitalization, institutionalization, and death when compared to people who fall less than two timer per year, or non-multiple fallers [14]. Hip pain, LBP, and multisite joint pain have been shown to individually increase fall risk [15–18]. This evidence, in conjunction with the known adverse effects of hip OA on functional decline and fall risk [7, 11, 12], suggests that hip OA signs and symptoms may further predispose older adults with CLBP to falling. To alleviate patient and public health burdens, it is imperative to investigate if coexisting hip OA signs and symptoms contribute to fall risk in older adults with CLBP.

To our knowledge, no study has assessed the extent to which hip joint symptoms affect risk of falls in older adults with a primary complaint of CLBP. The purpose of this study was to determine if hip OA signs and symptoms were associated with falls in older adults with CLBP. We hypothesized that an increased number of hip OA signs and symptoms would correspond to elevated fall risk over 1-year follow-up.

## Methods

### Study overview

This is a secondary analysis of data from a 12-month prospective study that included 250 generally healthy community-dwelling older adults between the ages of 60 and 85 years with CLBP who could ambulate independently with or without a single point cane (Hicks GE, Pohlig RT, Coyle PC, Sions JM, Weiner DK, Pugliese JM, et al.: Classification of geriatric low back pain based on hip characteristics with a 12-month longitudinal exploration of clinical outcomes: Findings from Delaware Spine Studies, submitted). CLBP status was characterized by research standards for this patient population [19]: LBP intensity ≥ 3/10, LBP frequency on four or more days of the week, LBP duration ≥ 3 months, and LBP that negatively impacts daily function. Participants were ineligible if they had lower extremity pain that was greater in intensity than their LBP, non-mechanical spinal conditions such as ankylosing spondylitis, current fractures in the back or hip, a progressive neurological diagnosis, or a terminal illness. Data for the parent study was collected in-person at baseline as well as 3 months, 6 months, and 12 months post-baseline in the University of Delaware’s Clinical Research Evaluation Laboratory; for this investigation, baseline clinical evaluation data and falls data throughout a12-month follow-up were utilized. The study was approved by the University of Delaware’s Institutional Review Board and was conducted in accordance with the Declaration of Helsinki. All participants signed an informed consent form.

### Baseline characteristics

Participants reported their age, sex, race, and fall history from the year prior to baseline. Height and weight were measured in centimeters (cm) and kilograms (kg), respectively, and converted to body mass index (BMI). Separate composite pain measures were created for the both hip and low back regions by averaging three pain intensity ratings for each area (0 = none, 10 = worst pain imaginable): highest and lowest pain over the last 24 h, and current pain at the time of baseline evaluation. The Activities-Specific Balance Confidence Scale (ABC-16) and Quebec Back Pain Disability Scale (QBPDS) are reliable and valid measures that were completed at baseline to obtain self-perceived balance confidence [20] and LBP-related disability [21], respectively. A detailed list of brand and generic medication names was used to obtain medication usage; medications from the benzodiazepine drug class were grouped as “anxiolytic medications” and summed together. Balance confidence [22, 23], LBP-related disability [24], and benzodiazepine usage [25] have previously been associated with elevated fall risk in older adults; as such, they were identified as covariates.

### Hip OA impairment burden

Hip OA signs and symptoms were assessed at baseline using the ACR guidelines: presence of hip pain, hip internal rotation (IR) range of motion (ROM) ≥ 15°, pain exacerbation with passive hip IR ROM, and morning stiffness lasting ≤ 60 min upon waking [8]; this subset of ACR criteria has been found to be prevalent among older adults with CLBP [7]. Hip pain was ascertained by asking participants, “Do you have pain in your groin/buttock/thigh?”, with possible responses of “yes” or “no”. Morning stiffness was determined by asking participants, “Do you have morning stiffness in your hip?”; if participants answered “yes”, they were queried, “Does it last less than or equal to 60 minutes?”. Both hip IR variables were collected during a clinical examination of passive ROM; the magnitude of ROM was measured in prone with an inclinometer [26, 27], while pain exacerbation during examination was defined as the onset of “groin, lateral thigh, or buttock pain”. The presence of hip pain and morning stiffness upon waking were coded as dichotomous variables (0 = no, 1 = yes), whereas hip IR range of motion and pain variables were collected for both hips and coded accordingly (0 = absent, 1 = unilateral, 2 = bilateral). ACR criteria were measured and summed to create a burden variable (hereafter referred to as hip OA impairment burden) with a maximum possible score of 6, with more criteria indicating a higher likelihood of hip OA [8]. All participants were > 50 years old, so that criterion was not included in the calculation.

### Falls outcome

A fall was operationally defined as any accidental or unintentional event in which contact was made with a lower surface or the ground [28]. Fall incidence was monitored via monthly fall calendars, which are the preferred method for prospective studies [29, 30]. At baseline, 3-month, and 6-month visits, participants were provided with a sufficient number of falls calendars and pre-stamped envelopes to account for time between in-person visits; participants were asked to mail their falls calendars to laboratory staff on a monthly basis. To prevent lapses in falls data, participants also received monthly follow-up phone calls to encourage timely return of fall calendars and to document any unreported falls that occurred in the previous month. On the fall calendars, participants were instructed to record an “F” on each day a fall occurred and an “N” on each day a fall did not occur. Outstanding calendars from prior follow-up time points were collected at the 12-month assessment visit. Falls were summed across the participants’ monthly calendars and follow-up calls. In the event that both the fall calendars were missing and the phone screens were incomplete, the falls data for that participant was excluded. If participants withdrew or died before study completion, that data was excluded as well. This adjudication of fall counts was completed prior to processing of all baseline data.

### Data analysis

**Fig. 1:**
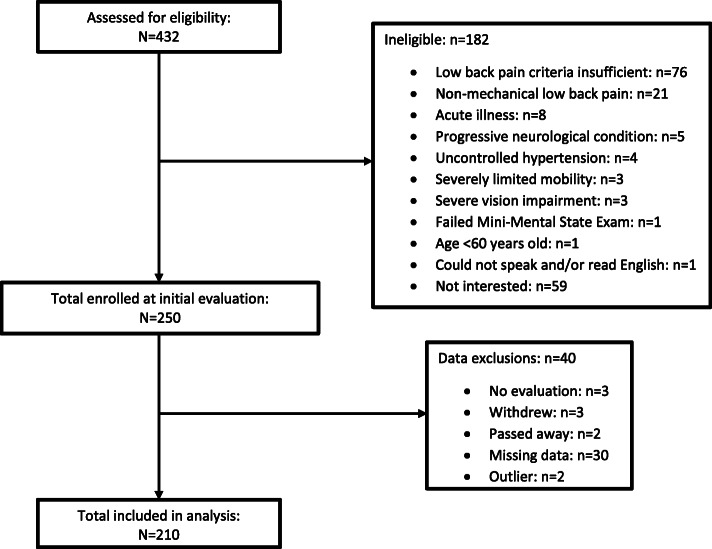
Study flow and participant inclusion process diagram

All analyses were completed on IBM SPSS Statistics versions 24 and 25 (SPSS, Inc. Armonk, NY). Only participants with complete falls data over 12 months were included (*n* = 212 of 250); two outliers in fall counts were removed from the sample based on the distribution of reported falls, leaving 210 for analysis (Fig. 1). Skewness and kurtosis were assessed for continuous variables to confirm compliance with parametric assumptions. Differences between included and excluded participants were assessed via chi-square and independent *t*-tests for nominal and continuous variables, respectively, and Mann-Whitney *U* tests for ordinal or non-normal variables. The prevalence of hip OA impairment burden in the study sample was assessed with frequencies. Differences in hip OA impairment burden between non-multiple and multiple fallers were assessed with Mann-Whitney *U* tests. The relationship between the number of falls and hip OA impairment burden was modeled via a Generalized Linear Model with Poisson distribution and log link function. To further assess if hip OA impairment burden was associated with the clinically relevant outcome of falling two or more times within a year [14], a binary logistic regression was performed as a secondary analysis. Both models adjusted for seven covariates: sex, age, BMI, anxiolytic use, balance confidence, LBP-related disability, and prior fall history. Bivariate correlation matrices were utilized to assess for multicollinearity among the covariates and hip OA impairment burden; cutoffs for bivariate correlation values were < 0.80. Significance was set to *p* < 0.05 for all analyses.

## Results

Excluded participants had significantly higher average LBP intensity and LBP-related disability compared to included participants, who had mild to moderate LBP intensity and LBP-related disability ratings (see Table 1). On average, included participants had moderate to high levels of balance confidence [31]. Over a 12-month span, 89 participants (42.4%) fell a total of 219 times. Among participants who fell, 50 (56.2%) fell more than once for a median of two and interquartile range of two (range from 1 to 12). Multicollinearity was not present among covariates and hip OA impairment burden (all *r* < 0.63).

**Table 1:** Included versus excluded participant characteristics

	Included (*N* = 210)	Excluded (*N* = 40)
	Mean ± SD or *N* (%)	
Sex: female	106 (50.5%)	22 (55.0%)
Race: white	181 (86.2%)	32 (80.0%)
Age (years)	69.85 ± 6.74	68.97 ± 7.38
BMI (kilograms/meters^2^)	29.37 ± 5.57	29.76 ± 6.42
Average LBP intensity (0–10)	2.96 ± 1.40	3.68 ± 1.67*
Average hip pain intensity (0–10)	1.72 ± 1.72	2.25 ± 1.89
QBPDS (0–100)	27.08 ± 16.21	34.66 ± 17.36*
ABC-16 (0–100)	80.85 ± 18.19	73.83 ± 22.32
Anxiolytics	0.08 ± 0.28	0.13 ± 0.33
Hip OA impairment burden (0–6)	3.07 ± 1.29	3.15 ± 1.33†
Prior falls	1.08 ± 2.84	1.08 ± 2.34†

Note: Categorical values are presented as total number (% of sample), while continuous values are presented as mean (± SD)

Abbreviations: *BMI*, body mass index; *LBP*, low back pain; *QBPDS*, Quebec Back Pain Disability Scale; *ABC-16*, Activities-Specific Balance Confidence Scale

*p < 0.05; † = distributions were non-normal, reported *p* value is from Mann-Whitney test

**Fig. 2:**
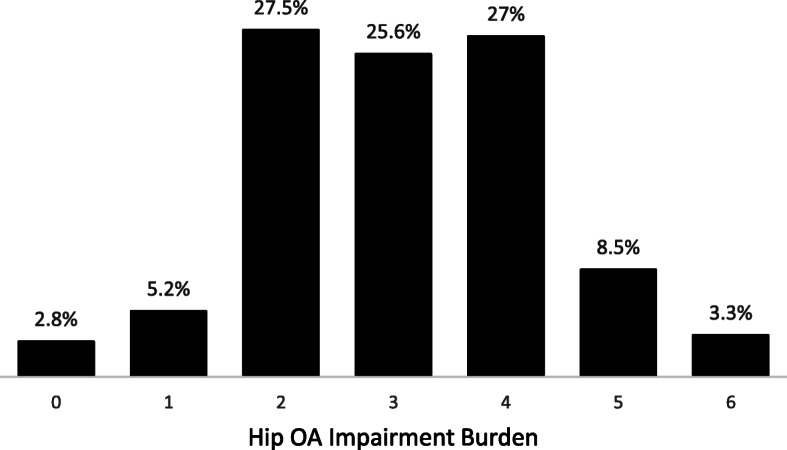
Frequency distribution of hip OA impairment burden

One or more hip OA signs and symptoms were present in 97.1% of our study population (Fig. 2). Hip IR ROM variables were the most common criteria present, with positive measurements in > 80% of participants; presence of hip pain (68.6%) and morning stiffness upon waking (43.3%) were also prevalent, while pain with passive hip IR ROM was less common (< 15%). Compared to non-multiple fallers, multiple fallers had significantly greater hip OA impairment burden (*p* < 0.05).

Associated relative risks (RR) and 95% confidence intervals (CI) are presented in Table 2. The overall generalized linear model was statistically significant (*p* < 0.005). After adjusting for covariates, hip OA impairment burden was significantly associated with fall risk (*p* = 0.001, RR = 1.23, 95% CI: 1.09–1.38); the RR corresponds to a 23% increase in the risk of falling for each additional ACR criterion present. Hip OA impairment burden was also associated with significantly increased odds of falling two or more times after adjusting for the covariates (*p* < 0.05, odds ratio = 1.41, 95% CI 1.01–1.95), such that a 1-unit increase in hip OA impairment burden corresponds to a 41% increase in the odds of falling multiple times within a year.

**Table 2:** Poisson regression model risk ratios

Variable	RR (95% CI)	*p* value
Age	1.01 (0.99–1.04)	0.190
Sex (ref = female)	1.04 (0.76–1.42)	0.815
BMI	1.00 (0.98–1.03)	0.839
Anxiolytics	2.39 (1.70–3.36)	< 0.001*
ABC-16	1.00 (0.99–1.01)	0.717
QBPDS	0.99 (0.98–1.00)	0.148
Prior falls	1.08 (1.05–1.11)	< 0.001*
Hip OA impairment burden	1.23 (1.09–1.38)	0.001*

Abbreviations: *RR*, risk ratio; CI, confidence interval; *BMI*, body mass index; *QBPDS*, Quebec Back Pain Disability Scale; *ABC-16*, Activities-Specific Balance Confidence Scale

*p < 0.05

## Discussion

Fall incidence over a 12-month period was considerably higher in this cohort of older adults with chronic low back pain (42.4%) compared to national estimates of 28.7% for this age group [13]. Hip OA signs and symptoms were highly prevalent and independent risk factors for falls in this cohort, such that each increase in hip OA impairment burden was associated with a successively greater risk of falling. Furthermore, greater hip OA impairment burden was associated with falling two or more times throughout study follow-up, which contextualizes the risk that is posed by elevated hip OA impairment burden in older adults. Our hypotheses were therefore supported. These findings are novel, as no study to date has investigated the additive effect of hip OA signs and symptoms on fall risk among older adults with CLBP.

Our findings are consistent with prior evidence that ACR criteria for hip OA are common in older adults with CLBP [7], and add to the body of literature that links hip OA signs and symptoms to greater fall risk in older adults [11, 12, 18]. Given that the intent of our study was to preliminarily assess if ACR hip OA criteria independently increased fall risk in older adults with CLBP, we cannot speculate on which criteria are driving this relationship. Sturnieks et al. [12] established that arthritis group membership, which significantly increased fall risk in older adults, was determined by neuromuscular characteristics more so than pain via discriminant function analysis. Munch et al. [18] demonstrated that self-reported hip pain was a risk factor for falls in older men independent of covariates, including osteoarthritis. Based on prior evidence, it is reasonable to infer that both rheumatologic [12] and nociceptive pain [18] characteristics contribute to the deleterious effects of hip OA signs and symptoms. Future investigations should aim to delineate which facets of hip OA signs and symptoms are contributing most to poor health outcomes in older adults with and without coexisting pain conditions.

Fall prevalence data from our study suggests that CLBP may increase fall risk. This finding aligns with work from Marshall et al. [16, 17], wherein back pain and LBP independently elevated fall risk in a subsample of the Osteoporotic Fractures in Men Study (MrOS) cohort and women from the Study of Osteoporotic Fractures (SOF) cohort, respectively. The cumulative weight of prior evidence plus our findings suggests that hip impairments compound fall risk in older adults; those with coexisting CLBP are at even further risk of falling.

Recent findings from Hicks et al. [7] showed that performance tests predictive of mobility decline (i.e., repeated chair rise and stair-climbing tests) were significantly worse among CLBP participants with coexisting hip impairments. Poorer performance on mobility tests increases an older adult’s risk for multiple falls [32]. Mobility decline is part of the pathway from health to adverse health outcomes such as falling; our results, in conjunction with prior evidence of susceptibility to mobility decline [7, 14, 32], establish that coexisting hip impairments increase fall risk and overall vulnerability of older adults with CLBP.

Our findings of increased fall risk secondary to higher hip OA impairment burden partially diverges with prior evidence. Leveille et al. [15] reported that back pain in combination with other pain sites does not affect fall risk in older adults. In the MrOS and SOF cohorts, hip pain prevalence incrementally increased with back pain and LBP severity; however, hip pain was not an effect modifier of the relationship between back pain and falls [16, 17]. It is possible that the results of these studies differ from our findings due to differences in study populations and methods of ascertaining pain. Clinical hip OA signs and symptoms may capture information that is unique from dichotomous classifications of hip pain, and as such may differentially affect prospective fall risk. Further, our criteria for CLBP are representative of a well-defined clinical population rather than a spectrum of LBP characteristics; risks and health outcomes for CLBP are distinct from other areas of back pain (i.e., thoracic) and non-chronic LBP.

The findings of the present study have clear clinical implications for older adults with CLBP. When combining the elevated fall risk from CLBP alone with the additional fall risk due to concomitant hip impairments, it becomes clear that healthcare professionals should screen older patients with CLBP for hip impairments as well as overall fall risk, and appropriately intervene. While further evidence is required, it seems that improving the symptoms associated with hip impairments may benefit older adults by reducing both fall risk and functional limitations. Therefore, plans of care with targeted treatment strategies should be considered for this important population.

This study has considerable strengths, including the sample size, use of fall calendars [29], adjustment for an array of well-reported risk factors, and a robust regression model. However, the results should be viewed in light of some limitations. Observational studies preclude the ability to make causative claims. Additionally, the studied cohort had mild self-reported back and hip pain and moderate LBP-related disability, which may limit generalizability to populations with more severe pain or disability. Further, given that excluded participants had significantly higher average LBP ratings and QBPDS values, our reported association between hip-related burden and risk of multiple falls may be underestimated. Finally, cutoffs for multicollinearity diagnostics are not universally agreed upon and may not entirely detect instances of multicollinearity [33], so the results should be interpreted with caution.

## Conclusion

Higher burden of hip OA signs and symptoms as identified by ACR criteria independently increased the risk of falls among older adults with CLBP. These results are an extension of prior evidence; LBP and hip pain have previously been established as independent risk factors for falls, and together are associated with reduced performance in repeated chair rise and stair-climbing tasks. The findings suggest that hip pain should be comprehensively evaluated in older adults who present with CLBP. Given that CLBP and concomitant hip OA signs and symptoms predispose older adults to falls, preventative intervention strategies should be considered in plans of care. Future studies should address how particular intervention strategies, such as hip-focused treatment, impact mobility and fall risk.

## Data Availability

The data analyzed during this study are presented in this article. Other data for the current study are not publicly available in order to maintain the confidentiality and anonymity of study participants; data may be available from the corresponding author upon reasonable request.

## Abbreviations

LBP: Low back pain
CLBP: Chronic low back pain
ACR: American College of Rheumatology
OA: Osteoarthritis
BMI: Body mass index
IR: Internal rotation
ROM: Range of motion
ABC-16: Activities-Specific Balance Confidence Scale
QBPDS: Quebec Back Pain Disability Scale
MrOS: Osteoporotic Fractures in Men Study
SOF: Study of Osteoporotic Fractures
RR: Risk ratio
OR: Odds ratio
